# Work Stress, Coping Strategies, and Health-Related Quality of Life among Nurses at an International Specialized Cancer Center

**DOI:** 10.31557/APJCP.2021.22.9.2995

**Published:** 2021-09

**Authors:** Majeda A Al-ruzzieh, Omar Ayaad

**Affiliations:** *King Hussien Cancer Center, Amman, Jordan. *

**Keywords:** Work Stress, Coping Strategies, Health-related Quality of Life, Nurses, Specialized Cancer Center

## Abstract

**Objective:**

The main objective of this study is to identify work stress, coping strategies, and health-related quality of life and the relationship between them among oncology nurses.

**Methods:**

A cross-sectional design was conducted at King Hussein Cancer Center. A convenience sampling technique was used to select 446 nurses. A self-administered questionnaire was utilized using three scales: the Work Stressor Inventory for Nurses in Oncology, Revised Ways of Coping Checklist, and Research and Development 36-Item for Health Survey.

**Results:**

The results showed that the levels of work stress (2.61/5), using coping strategy scale (1.59/4), and health-related quality of life scale (50.54/100) were moderate. The total mean value of the work stress scale had a significant positive correlation with the total mean value of the coping strategy scale (r=0.322*, p < 0.05) and a significant negative correlation with health-related quality of life. Moreover, there is no significant correlation between the total mean value of the coping strategy scale and the health-related quality of life scale (r=0121, p >0.05). Age and years of experience were negatively correlated with health-related quality of life (r=0.217 and 0.182 respectively, p < 0.05).

**Conclusion:**

Oncology nurses had a moderate level of work stress, coping strategy scale, health-related quality of life scale. Work stress has a significant correlation with using coping strategies and health-related quality of life among oncology nurses. Proper training regarding effective coping strategies is required. More studies are recommended to examine work stress, coping strategy, and health quality of life among oncology nurses.

## Introduction

Work stress is defined as the harmful emotional and physical reactions that occur when employees do not meet work needs and requirements (Canadian Mental Health Association, 2018). Work stress occurs in all professions and work settings, including healthcare where nurses work. Globally, the prevalence of work stress among nurses varies between 9.2% and 68.0% (Dagget et al., 2016). 

Many studies indicated that oncology nurses work in a highly stressful environment due to many factors. These factors are related to workload; co-worker stress; facing patients’ suffering, death, and dying; and handling the requirements of patients and their relatives (Borteyrou et al., 2014; Gómez-Urquiza et al., 2016; Ko and Kiser-Larson, 2016; Naholi et al., 2015; Wahlberg et al., 2016). 

Wahlberg et al. found that stress among oncology nurses working in inpatient units scored 8.2 out of 10 where job workload, organization management and system, and dealing with dying patients were perceived as frequent high stressors (Wahlberg et al., 2016). Naholi et al., (2015) found that job workload, dealing with dying patients, and inadequate preparation were the highest frequent stressors among new oncology nurses. 

Many studies indicated that work stress could negatively affect nurses’ well-being and health outcome (Adriaenssens et al., 2017; Giarelli et al., 2016; Sarafis et al., 2016). It harms the health-related quality of life, including physical, social, emotional functions, mood, or thinking. The impacts on physical functions include headache; chest pains; increased blood pressure, heart rate, fatigue, and insomnia; muscle tension or pain; weakened immune system; and high blood sugar. The impacts on mood or thinking include mood swings, hypersensitivity, forgetfulness, irritability, defensiveness, anxiety, restlessness, and anger (Canadian Mental Health Association, 2018; Sarafis et al., 2016).

Consequently, these impacts negatively affect oncology nurses; they make errors in judgment and tasks, have a decreased nursing satisfaction, increased turnover and burnout (López-López et al., 2019; Meyer et al., 2015), thus decreasing performance and productivity, neglecting responsibilities, becoming distracted, and reacting inadequately during routine activities (Adriaenssens et al., 2017; Wazqar et al., 2017). All these impacts may be negatively affecting patient outcomes. 

However, work stress impacts are different among individuals, depending on adapted coping strategies and demographic characteristics such as age, years of experience, and working area (Mazzella Ebstein et al., 2019; Wazqar et al., 2017). Coping strategies are defined as “an action, a series of actions, or a thought process used in meeting a stressful or unpleasant situation or in modifying one’s reaction to such a situation”(American Psychological Association, 2018). 

Coping strategies could be categorized into eight domains: wishful thinking, problem-focused coping, avoidance, self-blame, counting of one’s blessings, blame of others, seeking social support, and religiousness (Choi et al., 2017; Ko and Kiser-Larson, 2016; Vitaliano et al., 1985; Wahlberg et al., 2016). 

A cross-sectional study was conducted in 2016 aimed to identify coping strategies to address stressors among nurses. Around 395 nurses filled the Ways of Coping scale. The results showed that problem-focused strategies were commonly used by head nurses, postgraduate nurses, and high experience nurses. Denial and avoidance strategies were commonly adopted in Intensive Care Unit nurses. Emotionally focused and blamed self-related strategies were adopted mainly by females (Zyga et al., 2016). Among oncology nurses, avoidance-oriented coping was the highest perceived coping strategy (Wahlberg et al., 2016), while verbalizing, taking time for self, and exercising or relaxing were the most used tactics (Ko and Kiser-Larson, 2016; Wahlberg et al., 2016). 

The evidence about stress, coping, and their effects on oncology nurses is considered limited, and considerable studies are needed to develop oncology nursing literature (Wazqar et al., 2017). This study is considered the first comprehensive study conducted in an oncology setting that discusses work stress, its coping, and health-related life, the relationships between them among oncology nurses. 

For this reason, the results aim to provide baseline information regarding work stress, coping strategies, and health-related quality of life in oncology nurses. Nursing researchers may utilize this information as baseline data for future work by health managers in oncology settings to establish the required projects and programs to improve health-related quality of life and reduce stress among oncology nurses. 

The main purpose of this study is to identify work stress, coping strategies, and health-related quality of life and the relationships between them among nurses in an international specialized cancer center. Moreover, the study elaborates how the Magnet designation and culture may play essential roles in decreasing work stress, guiding coping strategies, and improving health-related quality of life. Many studies indicate the role of Magnet culture and designation in improving patient and nurse outcomes (Speroni et al., 2021). The roles may be mediated by effective, shared governance and a professional practice model, which are the main requirements for the Magnet accreditation that enhance the working environment (Ayaad et al., 2018, Al-Ruzzieh and Ayaad 2020).

## Materials and Methods

*Design and Setting*

The study used a cross-sectional design. A cross-sectional study is generally used to investigate certain phenomena and relationships between study variables. It is relatively inexpensive and faster compared to other designs.This study was conducted in King Hussein Cancer Center (KHCC), Jordan. KHCC is a not-for-profit and one of the biggest cancer centers in the Middle East region that provide comprehensive cancer care with a 350-bed capacity. Around 1200 nurses work at KHCC. 

The nursing department earned a Magnet designation in 2019. Throughout the world, the Magnet^®^ Recognition Program recognizes hospitals in which nursing executives have effectively aligned their strategic nursing goals to improve the organization’s patient outcomes. The Magnet Recognition Program serves as a road map to nursing excellence, which is beneficial to the entire hospital. Magnet Recognition signifies continued education and growth throughout the nurses’ careers, resulting in increased autonomy and satisfaction (American Nurses Credentialing Center, 2021).

*Sample*

The sample was selected using a convenience sampling technique. This technique is used to capture the highest number of participants in a short time (Cooper and Schindler, 2014). The inclusion criteria for selecting the sample included frontline nurses who worked at KHCC at sampling. The minimal sample size for this study was 306 (confidence interval 95% and 5% margin of error)(Cooper and Schindler, 2014). 

*Instrument*

The data was collected using a self-administered questionnaire. This questionnaire started with items related to demographic information, including gender, years of experience, working unit, and educational level (bachelor’s and master’s degrees). 

*The Work Stressor Inventory for Nurses in Oncology (WSINO)*

The Work Stressor Inventory for Nurses in Oncology (WSINO) was used to identify work stressors nurses may face during work. This questionnaire has 51 items covering five categories: workload, dealing with suffering, co-worker stress, death and dying, and dealing with patients and relatives. Using a 5-point Likert scale (1: the stressor has never happened, 5: indicated the stressor frequently happens). The Cronbach’s coefficient of the items of this scale ranged from 0.83–0.92. The five rotated factors accounted for 48.19% of the total variance. The loading factor analyses for all items ranged from .39-89 (Borteyrou et al., 2014).

*The Revised Ways of Coping Checklist (RWCCL)*

RWCCL was used to determine coping strategies used by oncology nurses. RWCCL includes 57 items covering eight types of coping strategies: wishful thinking, problem-focused coping, avoidance, self-blame, counting of one’s blessings, the blame of others, seeking social supports, and religiousness. A four-point Likert scale (0-3) was used, where 0 indicates ‘never used’, and three indicates ‘regularly used’. The Cronbach’s coefficient of the items in this scale ranged from 0.73–0.88, and the loading factor analyses for all items were above .39 (Vitaliano et al., 1985).

*Research and Development (RAND) 36-Item Health Survey (Version 1.0)*

The Research And Development Corporation developed RAND, a 36-Item Health Survey (Version 1.0). It was used to measure the health-related quality of life among oncology nurses. It covers eight health concepts: physical health, limitations due to physical problems, limitations due to emotional problems, energy, emotional well-being, bodily pain, social functioning, and general health. Different scoring scales were used in this survey. However, the total value for each statement was calculated out of 100, where 100 was the highest positive level (Ware and Sherbourne, 1992). Many studies confirmed the reliability and validity of the survey (McHorney et al., 1993, McHorney et al., 1994). 

*Data Collection*

We identified potential participants by reviewing the staffing database against the eligible criteria. The questionnaire was uploaded as an internet-based form to make the data collection process easy. It was combined with a cover letter and was sent to all potential participants via email. The data collection was conducted in August 2020.

*Data Analyses*

Statistical Package for the Social Sciences (SPSS) version 20 was used to analyze the data. Descriptive analysis, including frequency, percentage, mean, and standard deviation, was used to describe participants’ demographic, mean value of working stress, coping strategies, and health-related quality of life. 

The total mean value was calculated for each study variable, which means the sum of all items’ scores for all participants divided by the number of participants multiplied by the number of items. The total mean value described the level of work stress (out of five), coping strategies stressor (out of four), and health-related quality of life stressor (out of 100) among participants. 

The relationships between level of stressor, coping strategies, and health-related quality of life, and between the total mean value of scales with nurses’ age and years of experience were calculated using correlation coefficient (r). P-value was considered significant at .05. Accordingly, the correlation was considered significant if the p-value was less than .05.

## Results

*Participants Demographics*

As Table 1 indicates, the sample consisted of 446 frontline nurses with a mean age of 28.28 years (SD=5.89) and a mean experience of 3.95 years (SD= 5.41). Most of them were female (282, 63.2%) and had a bachelor’s degree (403, 63.2%). Most of the participants worked in outpatient and ambulatory units (158, 35.43%) and adult medical-surgical units (113, 25.34%).

*Mean values of Work Stress, Coping Strategy, and Health-related Quality of Life Scales*

**Figure 1:**
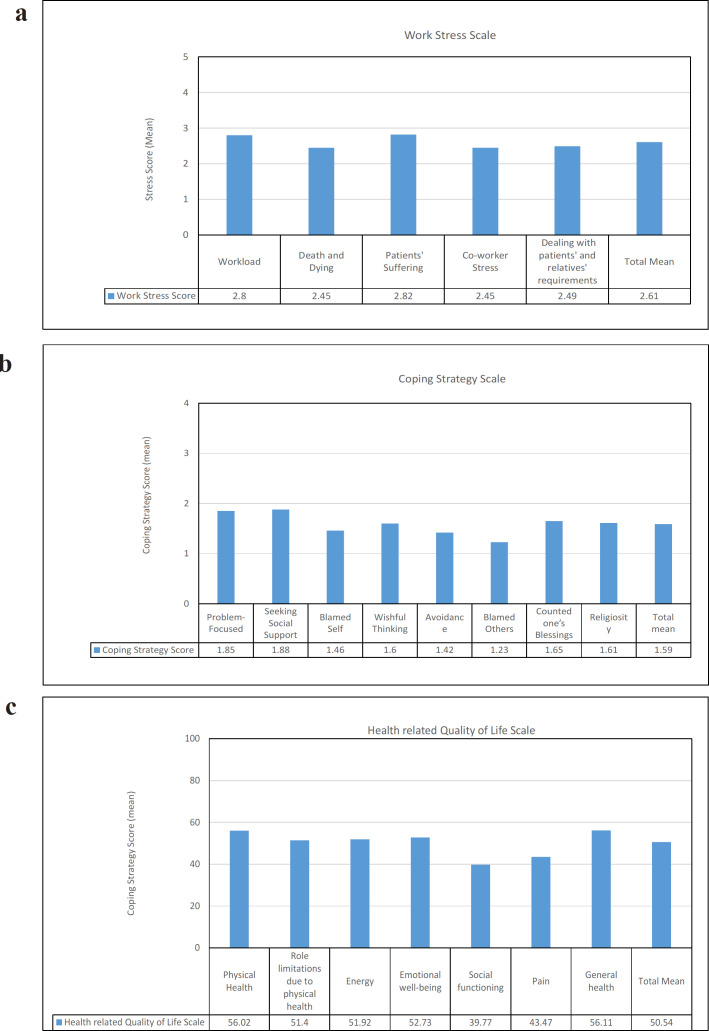
Mean Values of Work Stress, Coping Strategy, and Health-Related Quality of Life Scales

Table 2 and Figure 1 presents the mean values for study scales. The total mean value of work stress was 2.61 (SD=0.61). The highest work stressor categories were related to patients’ suffering (mean=2.80; SD = 0.77) and those related to workload (mean = 2.80; SD = 0.70). The total mean value for the coping strategy scale was 1.59 (SD=0.24). The most used coping strategies were “seeking social support” (mean=1.88; SD = 0.71) and problem-focused coping (mean=1.85; SD = 0.61), while the least used coping strategies were blaming others (mean=1.23; SD = 0.65) and avoidance (mean=2.80; SD = 0.77). The total mean value for health-related quality of life was 50.54 (SD=14.63). The general health (mean=56.11, SD=16.10) and physical health (mean=56.02, SD=27.43) dimensions were the best-perceived dimensions of health-related quality of life.

*Correlation between Work Stress Scale and Coping Strategy Scale*

Table 3 presents the correlation of the work stress scale with the coping strategy scale. The results show that the total mean value of the work stress scale and its subtypes had a significant positive correlation with the total mean value of the coping strategy scale (r=0.322*, p < 0.05) and its subtypes (p < 0.05). This result indicates that increasing the perception of work stressors increased the use of coping strategies. 

*Correlation between Work Stress Scale and Health-Related Quality of Life*

Table 4 presents the correlation of the work stress scale with the health-related quality of life scale. The results show that the total mean value of the work stress scale significantly negatively correlated with health-related quality of life (r=-.214, p < 0.05) and its subtypes, except physical health (r= -.008, p >0.05) and role limitation due to physical problems (r= -.091, p >0.05). This result indicates that increasing the perception of work stressors decreased the health-related quality of life among oncology nurses. Likewise, the results indicate that all work stress subtypes were significantly correlated with the total mean value of the health-related quality of life scale except patients’ suffering (r=-0.087, p <0.05). 

*Correlation Coping Strategy Scale and Health-related Quality of Life Scale*

Table 5 presents the correlation of the coping strategy scale with the health-related quality of life scales. The results indicate no significant correlation between the total mean values of the coping strategy scale and the health-related quality of life scale (r=0.121, p >0.05). On the other hand, the total mean value of the coping strategy scale only had a significant positive correlation with the physical health dimension for health-related quality of life (r=0.096*, p <0.05). 

However, it was indicated that a significant (positively or negatively) correlations between all coping strategies and the total mean value of health-related quality of life (p < 0.05) except the religiosity strategy (r=0.005, p >0.05). The significant positive correlations were related to problem-focused, seeking social support, wishful thinking, and counted one’s blessings strategies, while the negative correlations were related to blamed self, blamed others, and avoidance strategies (p <0.05). 

*Work Stress Scale, Coping Strategy Scale and Health-related Quality of Life Scale According to Age and Years of Experience*

Table 6 indicates the correlation between the total mean value of the work stress scale, coping strategy scale, health-related quality of life scale, and age and years of experience. The results show a significant negative correlation between age and years of experience and health-related quality of life (r=0.217 and 0.182, respectively, p < 0.05).

**Table 1:** Participants Demographics

Demographic Characteristic	Results
Gender (N, %)	
Male	164 (36.8)
Female	282 (63.2)
Educational level (N, %)	
Bachelor	403 (90.4)
Postgraduate	43 (9.6%)
Age (Mean, SD)	28.28 (5.89)
Years of Experience (Mean, SD)	3.95 (5.41)
Working Unit Type (N, %)	
Adult Medical Surgical Units	113 (25.34)
Adult Intensive and intermediate care units	79 (17.71)
Bone Marrow Transplant units	59 (13.23)
Pediatric Unit	19 (4.26)
Pediatric Intensive care unit	18 (4.04)
Outpatient and Ambulatory Units	158 (35.43)
Total (N, %)	446 (100)

**Table 2:** Mean Values of Stress and Coping Strategy, and Health related Quality of Life Scales

Demographic Characteristic	Mean (SD)
Work Stress Scale (Five Likert scale)	
Workload	2.80 (.70)
Death and Dying	2.45 (.71)
Patients' Suffering	2.82 (.77)
Co-worker Stress	2.45 (.71)
Dealing with patients' and relatives' requirements	2.49 (.61)
Total Mean	2.61 (.61)
Coping Strategy Scale (Four Likert scale)	
Problem-Focused	1.85 (.61)
Seeking Social Support	1.88 (.71)
Blamed Self	1.46 (.71)
Wishful Thinking	1.60 (.65)
Avoidance	1.42 (.59)
Blamed Others	1.23 (.65)
Counted one’s Blessings	1.65 (.65)
Religiosity	1.61 (.73)
Total mean	1.59 (.24)
Health related Quality of Life Scale (Out of 100)	
Physical Health	56.02 (27.43)
Role limitations due to physical health	51.40 (35.74)
Role limitations due to emotional problems	52.91 (38.38)
Energy	51.92 (12.06)
Emotional well-being	52.73 (14.72)
Social functioning	39.77 (22.05)
Pain	43.47 (17.91)
General health	56.11 (16.10)
Total Mean	50.54 (14.63)

**Table 3:** Correlation between Means Values of Stress Scales and Coping Strategy Scales using Correlation Coefficient (r)

	Coping Strategy Dimensions	
Work Stressors	(r)	P value
Workload	1.670*	p < 0.05
Death and Dying	0.251*	p < 0.05
Patients' Suffering	0.294*	p < 0.05
Co-worker Stress	0.308*	p < 0.05
Dealing with patients' and relatives	0.274*	p < 0.05
Total	0.322*	p < 0.05
Problem-Focused	0.229*	p < 0.05
Seeks Social Support	0.153*	p < 0.05
Blamed Self	0.210*	p < 0.05
Wishful Thinking	0.283*	p < 0.05
Avoidance	0.298*	p < 0.05
Blamed Others	0.241*	p < 0.05
Counted one’s Blessings	0.280*	p < 0.05
Religiosity	0.268*	p < 0.05
Total	0.322*	p < 0.05

*Signifcant correlation

**Table 4:** Correlation between Stress Scales and Health Related Quality of Life Scales using Correlation Coefficient (r)

	Health related Quality of Life Dimentions	
Work Stressors	(r)	P value
Workload	0.285*	p < 0.05
Death and Dying	-0.156 *	p < 0.05
Patients' Suffering	-0.087	p > 0.05
Co-worker Stress	-0.201*	p < 0.05
Dealing with patients' and relatives	-0.186*	p < 0.05
Total	-0.214*	p < 0.05
Health related Quality of Life Dimentions	Work Stressors	
	(r)	P value
Physical Health	-0.008	p > 0.05
Role limitations due to physical health	-0.091	
Role limitations due to emotional problems	-0.194*	p < 0.05
Energy/fatigue	-0.179*	p < 0.05
Emotional well-being	-0.154*	p < 0.05
Social functioning	-0.103*	p < 0.05
Pain	-0.205*	p < 0.05
General health	-0.243*	p < 0.05
Total	-0.214*	p < 0.05

*Signifcant correlation

**Table 5:** Correlation Coping Strategy Scales and Health Related Quality of Life Scale using Correlation Coefficient (r)

Coping Strategy Dimensions	Health related Quality of Life Dimentions	
	(r)	P value
Problem-Focused	0.227	p > 0.05
Seeks Social Support	0.218*	p < 0.05
Blamed Self	-0.088	p > 0.05
Wishful Thinking	.104*	p < 0.05
Avoidance	-0.137	p > 0.05
Blamed Others	-0.178	p > 0.05
Counted one’s Blessings	0.122*	p < 0.05
Religiosity	0.005	p > 0.05
Total	0.0121	p > 0.05
Health related Quality of Life Dimentions	Coping Strategy Dimensions	
	(r)	P value
Physical Health	0.096*	p < 0.05
Role limitations due to physical health	0.01	p > 0.05
Role limitations due to emotional problems	-0.023	p > 0.05
Energy/fatigue	0.002	p > 0.05
Emotional well-being	0.014	p > 0.05
Social functioning	0.025	p > 0.05
Pain	-0.083	p > 0.05
General health	0.023	p > 0.05
Total	0.0121	p > 0.05

*Signifcant correlation

**Table 6:** Work Stress Scale, Coping Strategy Scale and Health Related Quality of Life Scale According to Age and Years of Experience using Correlation Coefficient (r)

Demographic Characteristic	Work Stress Scale		Coping Strategy Scale		Health related Quality of Life Scale	
	r	P value	r	P value	r	P value
Age	-0.033	0.497	0.002	0.957	0.217	0.00*
Years of Experience	-0.04	0.42	0.034	0.468	0.182	0.00*

*Signifcant correlation at level of aphla equals .05

## Discussion

The main purpose of this study is to identify work stress, coping strategies, and health-related quality of life and the relationships between them among oncology nurses. The total mean value of work stress was 2.61 (SD=0.61), which was considered acceptable compared to other studies. A previous meta-analysis study on the prevalence of work stress among nurses estimated it at 69%, which was considered moderate (Gheshlagh et al., 2017). Abdali Bardeh et al., (2016) indicated that around 95% of nurses perceived stress in their work as moderate and severe. Tuna and Baykal (2014) indicated that work stress among oncology nurses was moderate (Mean =7.98, SD=1.77). 

The highest work stressor categories were related to patients’ suffering (mean=2.80; SD = 0.77) and workload (mean = 2.80; SD = 0.70). Wazqar (2019) indicated that oncology nurses continuously feel attached and connected to patients with cancer due to the extended length of stay at hospitals. Moreover, many patients with cancer had worsened suddenly. For this reason, the experience of a deep sense of weakness and helplessness is elevated. On the other hand, the nurses’ shortage and high expectations of the oncology nurses are considered the main stressors that led to an increased perception of workload and physical and mental exhaustion. (Wazqar, 2019). 

However, it is expected that the nurses at Magnet hospitals may have lower stress levels due to the high feeling of nurse autonomy and satisfaction, due to the high organizational culture and environment, relationships and collaboration, training and development, pay and incentives, and the sufficiency of resources and facilities (Abuseif et al., 2018; Speroni et al., 2021). Many studies showed the role of adoption of shared governance, which is an essential component in magnet culture in improving the nursing work environment, in decreasing work stress, increasing satisfaction and commitment to the organization, and decreasing nurse turnover (Abuseif, and Ayaad, 2018; Ayaad et al., 2019; Speroni et al., 2021).

The total mean value for the coping strategy scale was 1.59 (SD=.24). The most used coping strategies were “seeking social support” (mean=1.88; SD = 0.71) and problem-focused coping (mean=1.85; SD = 0.61). The coping strategies used to handle stressors depend on the nurses’ beliefs, responsibilities, social skills, stress level, support, and available material resources (Schmidt et al., 2009). As many studies show, oncology nurses mostly use problem-focused coping such as acceptance, planning, problem-solving, and positive reappraisal, especially when the stressors are related to workload and job demand (Gomes et al., 2013; Rodrigues and Chaves, 2008; Wazqar, 2019). Likewise, seeking social support is generally utilized to compact emotional stressors such as patient suffering and feeling of helplessness (Umann et al., 2014; Wazqar, 2019). 

Magnet culture encourages nurse autonomy by adopting a shared governance model, which guides the decision-making process and enables nurses to share their ideas and views to solving problems in nursing (Ayaad et al., 2018; Speroni et al. 2021). This may explain why the “seeking social support” and problem-focused got the highest scores.

The total mean value of the health-related quality of life scale was 50.54 (SD=14.63). A study showed that the mean value health-related quality of life among nurses was around 45%, where the mean physical component summary score was 45.02%, and the mental component was 45.50% (Sarafis et al., 2016). Our setting showed that health-related quality of life dimensions with the highest mean values related to general health (mean=56.11; SD = 16.10) and physical health (mean=56, 02; SD = 27.43). A change in the score may be related to variations in the work environment and workload in different settings (Sarafis et al., 2016). 

Magnet culture encourages the nurse’s well-being by developing many initiatives and quality projects to enhance nursing well-being and quality of life through decreasing the stressors that affect health-related quality of life, such as workload and incidence of errors (Ayaad et al., 2019; Al-Ruzzieh and Ayaad 2020; Haroun et al., 2021).

The results showed that the total mean value of the work stress scale had a significant positive correlation with the total mean value of the coping strategy scale (r=0.322*, p < 0.05), which indicated that increasing the perception of work stressors increased the use of coping strategies. These results are consistent with many previous studies conducted in different hospital units in Iran, Hong Kong, China, Brazil, South Africa, USA, Malaysia, and Indonesia (Gomes et al., 2013; Rodrigues and Chaves, 2008; Wang et al., 2011; Wazqar, 2019). 

Many studies found that problem-focused strategies are generally more utilized than emotionally related strategies. However, it was indicated that high work stress is significantly correlated with using emotional-focus strategies such as avoidance, blaming self, wishful thinking, and ‘blaming others’ strategies. (Umann et al., 2014; Wang et al., 2011). Our results indicated that the patient suffering stressor was the highest and was correlated chiefly with problem-focused coping and counting one’s blessings. In contrast, the workload stressor, the second-highest, was primarily correlated with problem-focused coping and avoidance.

The results showed that the total mean value of the work stress scale significantly negatively correlated with health-related quality of life (r=-0.214, p < 0.05). This result indicated that increasing work stressors decreased the health-related quality of life among oncology nurses. This result is consistent with many previous studies (Adriaenssens et al., 2017; López-López et al., 2019; Meyer et al., 2015; Sarafis et al., 2016; Venugopal et al., 2020; Wazqar et al., 2017). However, our results showed a non-significant impact of stress on physical health and role limitation due to physical health, which may indicate the emotional impacts of stressors were higher than physical impacts. It could be due to the effectiveness of interventions that are performed in our setting to enhance and improve nurses’ well-being in terms of staffing, work environment, and quality improvement projects. 

Moreover, the results indicated no significant correlation between the total mean value of different coping strategies and the health-related life scale (r=0.121, p >0.05). It had a significant positive correlation with only the physical health dimension for health-related quality of life. The highest correlations with health-related quality of life were utilizing problem-focused coping and seeking social support strategies. These results are consistent with many previous studies (Chang et al., 2006; Gomes et al., 2013; Rodrigues and Chaves, 2008; Umann et al., 2014; Wazqar, 2019). Moreover, some emotionally focused strategies such as blaming self and others and avoidance strategies had a significant negative correlation with different health-related quality of life-related dimensions. This may stem from their role in delaying the stress instead of solving it.

Finally, the results showed a significant negative correlation between age and years of experience and health-related quality of life (r=0.217 and 0.182, respectively, p < 0.05). These results were expected due to the negative impact of age and years of experience on physical, social, and emotional well-being (CDC, 2018; Chang et al., 2006).

The study has many limitations, such as the variability of the nurses’ working areas, years of experience, professional levels, and the lack of some social characteristics of participants such as marital status, number of children, economic status, and place of residence. Moreover, the sample was selected using a convenience sampling technique in one oncology setting, which limited the generalizability of findings. The study did not indicate the role of the adoption of e-health in nursing practice, which could influence the results due to its role in enhancing nursing work and team effectiveness (Qaddumi et al., 2021, Al-Ruzzieh et al., 2020; Abu Sharikh, 2020).

In conclusion, oncology nurses had a moderate work stress level. The highest work stressor categories were related to patients’ suffering and workload. The most used coping strategies were ‘seeking social support’ and problem-focused coping, while the total mean value of the health-related quality of life scale was moderate.

The total mean value of the work stress scale had a significant positive correlation with the total mean value of the coping strategy scale. The problem-focused coping strategies were generally more utilized than the emotionally related strategies. Our results indicated that the patient suffering stressor was the highest and mainly was correlated with problem-focused coping, counting one’s blessings. The workload stressor, the second-highest, was primarily correlated with problem-focused coping and avoidance. The total mean value of the work stress scale had a significant negative correlation with health-related quality of life. 

## Author Contribution Statement

The authors confirm that both authors (Majeda A Al-Ruzzieh and Omar Ayaad) actively participated in study conception and design, data collection, analysis, interpretation of results, and draft manuscript preparation processes. All authors reviewed the results and approved the final version of the manuscript.
